# Patient experiences of engagement with care plans and healthcare professionals’ perceptions of that engagement

**DOI:** 10.1186/s12913-017-2806-y

**Published:** 2017-12-29

**Authors:** Mohamad Al-Tannir, Fahad AlGahtani, Amani Abu-Shaheen, Sawsan Al-Tannir, Isamme AlFayyad

**Affiliations:** Chairperson of Clinical and Applied Research Department, Research Center, King Fahad Medical City, P.O. Box 59046, Riyadh, 11525 Saudi Arabia

**Keywords:** Care plan, Engagement, Patient, Saudi Arabia

## Abstract

**Background:**

Although patient engagement is internationally recognized as a core quality indicator of healthcare systems, no report has yet explored patient engagement in Saudi Arabia. Thus, we explored patients’ experiences of engagement with healthcare services and assessed physicians’ and nurses’ perceptions of this engagement.

**Methods:**

We performed a cross-sectional study on patients and their family members admitted to either the rehabilitation or neurology department of King Fahad Medical City, Riyadh, Saudi Arabia. We also studied physicians and nurses involved in direct patient care in these departments. Two self-administered questionnaires were used to collect data on patients’ experiences of engagement with healthcare services and physicians’ and nurses’ perceptions of that engagement.

**Results:**

We recruited 36 patients and 46 family members, as well as 64 nurses and 36 physicians. About 73% of patients and family members felt that doctors and nurses engaged them in decision making regarding care plans; 80% felt that they were a partners in the treatment plans. Over one-third of physicians and nurses believed that patient engagement improved healthcare outcomes, and about 7% believed that patient engagement was unimportant or not extremely important. Responses of physicians and nurses differed significantly from those of patients and family members with regards to the extent of the patient–physician/nurse relationship, the perception of involvement, and the degree of partnership and shared leadership.

**Conclusion:**

We assessed patient experiences of engagement with health care service and physicians’ and nurses’ perceptions of that engagement. Most patients/family members reported good engagement. Although most physicians and nurses believed that patient engagement improved the healthcare outcomes, some believed that improving healthcare outcomes through patient engagement was not important or not extremely important.

## Background

Today, patient engagement is extensively and internationally recognized as important, and has gained growing policy attention, support by scientific evidence [1–6]. Patient engagement is considered a cornerstone of high-quality healthcare, improving health outcomes and reducing healthcare costs [7, 8]. Most Western countries have implemented formal governmental programs prioritize the “patient’s voice” and the “patient’s active role in their healthcare” [6, 9, 10].

The definitions of patient engagement vary considerably. Despite such variations, Coulter’s emphasized the importance of the relationship between patients and healthcare providers, who work together to “promote and support active patient and public involvement in health and healthcare and to strengthen their influence on healthcare decisions, at both the individual and collective levels” [11]. Carman et al. (2013) defined such engagement as patients, families, their representatives, and health professionals working in active partnership at various levels across the health care system direct care, organizational design and governance, and policy-making—to improve health and health care [3]. Graffigna (2013) defined patient engagement as a “process-like and multidimensional experience, resulting from the conjoint cognitive (think), emotional (feel), and conative (act) enactment of individuals toward their health management” [12].

The Institute of Medicine (IOM) has adopted patient-centered care as one of the six pillars of quality, and it considers that patient engagement is essential to motivate further research. The IOM has recommended that patients should receive access to health information and knowledge, enabling them to control healthcare decision making [13]. The cardinal rationale for engaging patients has its roots in the ethical principles of respecting the patient’s autonomy and promoting self-determination. However, patient engagement should not mean that patients bear the final responsibility for the safety of the care, which remains with the health care system’s [3, 14].

Many studies worldwide have described the importance of, and benefits affored by, patient engagement and its effect on the safety and quality of healthcare [15–20]. The benefits include improved patient adherence to care plan, better clinical outcomes, and increased satisfaction, and reductions in healthcare costs due to reduced hospitalization, decreased frequency of untoward outcomes, and fewer visits to doctors [6, 21].

Patient engagement with the nursing community is well recognized and is emphasized in healthcare settings and managing disease [6]. However, O’Leary et al. (2010) showed that only about one-third of hospital-surveyed patients were able to name one of their hospital physicians [22]. In Western countries, nurses have a deep-rooted tradition of patient engagement in medical treatment, and they are trained to foster patient autonomy, self-determination, and collaborative healthcare at all times [23]. The manner in which healthcare professionals interact with patients affects patient participation in their health care, which increases when healthcare professionals respond positively to patients’ needs and concerns and provide feedback to patients. Thus, as Carman et al. emphasized, patient engagement is a continuous process best implemented across all levels of a healthcare institution [3].

As physicians and nurses are at the frontline of patients care, Carman’s “Framework For Patient and Family Engagement in Health and Health Care” directs that patient enagagement should begin by incorporating patients’ values, perspectives, preferences, and experience in disease prevention, diagnosis, and treatment. Supporting patient engagement means that patients must be actively involved in care plans, communicating their goals, making shared decisions, and proactively managing their health. Moreover, physicians and nurses must help patients to communicate, and to understand and balance the risks and benefits of their healthcare choices. Physicians and nurses must promptly give patients, as much information as possible to aid in their understanding, and must involve the family and support persons [3].

Information about patients’ perspectives on engagement reveal their lived experiences and the extent of care provided by healthcare providers, which aid in future decision making. In addition, patient engagement surveys can be used to improve identified areas of weakness in the healthcare system by encouraging appropriate organizational decisions. Such information can also be used to hold physicians and nurses accountable. The patient Health Engagement Scale (PHE-scale) developed by Graffigna (2015) is a valid and reliable tool for evaluating patient engagement and its impact on the quality of care, health outcomes, and costs. The scale was developed after robust theoretical conceptualization and rigorous psychometric validation [12]. The Patient Activation Measure (PAM) is a valid and reliable scale for evaluating patient activation. This individualized tool can be used assess patients and develop care plans [24]. The Clinician Support for Patient Activation Measure (CS-PAM) was designed to assess clinicians’ knowledge and beliefs about patient self-management and activation. The measure adequately explores clinicians’ attitudes and beliefs regarding patient self-management [25].

Burns et al. (2014) reported that healthcare systems found it challenging to develop and implement engagement among patients, their families, healthcare providers, and healthcare administrators [26]. Although patient engagement seems to be very successful in Western countries, no relevant studies have addressed the situation in Saudi Arabia and throughout the region. Therefore, hospital managers require assistance in fostering patient engagement so that patients can participate effectively in their care.

Our study is valuable because we explore a unique culture in which family-centeric decision making influences patient autonomy [27]. Therefore, we assessed patients’ experiences of engagement with a tertiary hospital in central Riyadh, Saudi Arabia, and physicians’ and nurses’ perceptions of that engagement and compare this perception with patients experience of enagaement.

## Methods

### Study design

Following Institutional Review Board approval and after obtaining informed consent from all participants, we conducted a cross-sectional survey between February and August 2016 at King Fahad Medical City (KFMC), Riyadh, Saudi Arabia. The study sites were two departments that treat chronically ill patients, the rehabilitation and neurology departments.

### Participants and recruitment

We included and studied patients or their family members who agreed and consented to answer questions. We also included and studied physicians and nurses involved in direct patient care. Using a non-probability convenience sampling technique, two self-administered questionnaires were distributed to collect data.

### Data collection

The study questionnaires were developed after an in-depth literature review [20, 28–30]. Prior to the principal study, we conducted a pilot study with 10 physicians and nurse and 10 patients to assess the clarity and reliability of the instruments. Those enrolled in the pilot study were excluded from the final analyses. Data were collected by a trained research assistant, who explained the study to all participants. Before participation in the study, informed consent was obtained from all patients (or family members involved in the care plan if the patient was incompetent). The same research assistant approached all physicians and nurses working in the study settings.

The questionnaire for patients and family members consisted of two parts. The first part collected demographic data (patient age, gender, level of education, and the person completing the questionnaire, if it was not the patient). The second part assessed three domains. (i) The nature of the patient–physicians/nurses relationship was examined using two items: “The doctor/nurse addressed me and referred to me directly” and “The doctor/nurse introduced themselves and identified their role in my care.” Involvement was covered by two items: “The doctor/nurse explained my treatment plan to me” and “The doctor/nurse engaged my family and me in decision making regarding my care.” Partnership and shared leadership were similarly identified using two items: “I feel that I am a partner in my treatment and healthcare plan” and “I feel that my opinions and concerns matter to the doctor/nurse responsible for my care.” These questions were measured using a four-point Likert-scale as follows: “1 - never,” “2 - some of the time,” “3 - most of the time,” and “4 – all of the time.” The Cronbach’s alpha was 0.92.

The second questionnaire was addressed to physicians and nurses and consisted of three parts. The first part covered position and years of experience; the second part was an open-ended question exploring perceptions about patient engagement. Part three assessed perceptions of the nature of (i) the physician/nurse–patient relationship (addressing the patient directly and introducing yourself and your role in the patient’s care); (ii) involvement (advocating for patient and family involvement in decision making by actively listening to the patient’s concerns about the treatment plan); and (iii) partnership and shared leadership (always asking the patient his/her opinion about major healthcare treatment decisions and holding the belief that patient engagement improves healthcare outcomes). Responses were based on a four-point Likert-scale as follows: “1 - not extremely important,” “2 - not important,” “3 - important,” and “4 - extremely important.” Cronbach’s alpha was 0.89.

### Sample size calculation

We obtained data on the numbers of wards and beds, percentage occupancy rates, and the numbers of physicians and nurses. The sample frame included a neurology and a rehabilitation department (representative in-patient wards) and physicians and nurses who were in direct contact with patients.

1. Patient sample size: For an alpha value of 0.05, an estimated SD of 1.1, and a maximum difference of 0.5 at a power of 0.95, the total sample size should be at least 65.
2. Nurse and physician sample size: For an alpha value of 0.05, an estimated SD of 0.8, and a maximum difference. of 0.4 at a power of 0.95, the sample size should be at least 84.

### Statistical analysis

Data were analyzed using SPSS version 21.0 (SPSS, Inc., Chicago, IL, USA). Descriptive statistics were employed to describe quantitative and categorical variables. Pearson’s chi-squared test was used to compare the distributions of categorical variables. A *p*-value less than 0.05 was considered significant.

### Qualitative analysis

All open-ended responses from physicians and nurses were reviewed by two authors, who manually generated codes for the principal themes based on the patterns of responses. As the responses were many and varied, sub-categories were created. The code categories and subcategories were then examined by two other authors for review, refinement as necessary, and finalization. We were careful to respect and include all opinions given and to incorporate them in the coding subcategories. All responses were objectively summarized and recorded to eliminate any potential bias arising from subjective interpretation.

## Results

### Patient and family members’ demographic characteristics

In total, 82 patients and family members participated in the study (36 questionnaires completed by patients and 46 completed by family members); the response rate was 75%. The mean age of responders was 37.9 ± 18.98 years. Participating patients were older (40.86 ± 15.92 years) than their family members. Females comprised 56.1% of respondents (16 patients and 22 family members). Most respondents had been educated to the high school level. No statistically significant demographic difference was found between patients and family members (Table 1).

**Table 1:** Demographic characteristics of patients and family members. (*n* = 82)

Demographic variables	Patients	Family members	Total	*p*-value
Participant who completed the assessment	36 (43.9%)	46 (56.1%)	82	0.441
Age (mean ± SD)	40.86 ± 15.92	35.61 ± 24.88	37.9 ± 18.98	0.053
Gender				
Male	20 (55.6%)	24 (52.1%)	44 (53.7%)	0.761
Female	16 (44.4%)	22 (57.9%)	38 (46.3%)	
Level of Education:				
Illiterate	5 (13.9%)	4 (8.7%)	9 (11%)	0.377
Primary school	9 (25%)	14 (30.4%)	23 (28%)	
High school	18 (50%)	20 (43.5%)	38 (46%)	
University and above	4 (11.1%)	8 (17.4%)	12 (15%)	

### Physicians’ and nurses’ demographic characteristics

A total of 100 healthcare professionals responded (64 nurses and 36 physicians); the response rate was 67%. Nurses represented 64% of respondents; more than half were from the neurology department. Most responding physicians were neurologists. Twenty-seven nurses (42.2%) had less than 5 years of experience, and slightly more than one-third had 6–10 years of experience. Most physicians (61.1%) had less than 5 years of experience. No statistically significant demographic difference was evident between physicians and nurses (Table 2).

**Table 2:** Distribution of physicians’ and nurses’ demographic characteristics

	Position		Total	*p* value
	Nurse (*n* = 64)	Physician (*n* = 36)		
Area				
Neurology department	34 (53.1%)	23 (63.9%)	47 (47%)	0.651
Rehabilitation department	30 (46.9%)	13 (36.1%)	53 (53%)	
Years of Experience				
1–5	27 (42.2%)	22 (61.1%)	49 (49%)	0.062
6–10	23 (35.9%)	12 (33.3%)	35 (35%)	
11–15	6 (9.4%)	1 (2.8%)	7 (7%)	
16–20	2 (3.1%)	0	2 (2%)	
> 20	6 (9.4%)	1 (2.8%)	7 (7%)	

Table 3 shows patient and patient’s families’ experiences of engagement in care plans. About 79% of patients and families reported that physicians and nurses addressed and referred to them directly. In addition, about 78% reported that physicians and nurses introduced themselves and identified their roles in the care plan. Moreover, 73% indicated that doctors and nurses engaged them in decisionmaking regarding the care plan, and 80% felt that they were partners in the treatment plans.

**Table 3:** Distribution of patient and family members’ experiences of engagement in care plans

Questions	Not performed	Performed some of the time	Performed most of the time	Always performed
1. The doctor/ nurse addressed and referred to me directly	1 (1.2)	6 (7.3)	10 (12.2)	65 (79.3)
2. The doctor/nurse introduced themselves and identified their role in my care.	0	5 (6.1)	13 (15.9)	64 (78)
3. The doctor/nurse explained my treatment plan to me.	3 (3.7)	5 (6.1)	13 (15.9)	61 (74.4)
4. The doctor/nurse engage me and my family in the decision-making regarding my care.	2 (2.4)	8 (9.8)	12 (14.6)	60 (73.2)
5. I feel that my opinions and concerns matter to the doctor/nurse responsible for my care.	3 (3.7)	5 (6.1)	12 (14.6)	62 (75.6)
6. I feel I am a partner in my own treatment and healthcare plan.	2 (2.4)	6 (7.3)	8 (9.8)	66 (80.5)

Table 4 lists patients’ and their families’ perception of engagement in care plans by gender and by who performed the assessment; no statistically significant differences were evident. However, across all questions posed, males were more engaged in care plans than were females, family members were more engaged than patients.

**Table 4:** Patients’ and family members’ perceptions of engagement in care plans by gender and by who performed the assessment

Items	Level	Gender		*P*-value	Who performed the assessment		*P*-value
		Female (*n* = 38)	Male (*n* = 44)		Patients (*n* = 36)	Family members (*n* = 46)	
The doctor/ nurse addressed and referred to me directly	Not performed	1 (2.6%)	0	0.620	0	1 (2.2%)	0.312
	Performed some of the time	2 (5.3%)	4 (9.1%)		3 (5.6%)	3 (6.5%)	
	Performed most of the time	4 (10.5%)	6 (13.6%)		2 (5.6%)	8 (17.4%)	
	Always performed	31 (81.6%)	34 (77.3%)		31 (86.1%)	34 (73.9%)	
The doctor/nurse self-introduced and identified their role in my care	Not performed	0	0	0.957	0	0	0.557
	Performed some of the time	2 (5.3%)	3 (6.8%)		2 (5.6%)	3 (6.5%)	
	Performed most of the time	6 (15.8%)	7 (15.9%)		4 (11.1%)	9 (19.6%)	
	Always performed	30 (78.9%)	34 (77.3%)		30 (83.3%)	34 (73.9%)	
The doctor/nurse explains to me my treatment plan.	Not performed	1 (2.6%)	2 (4.5%)	0.660	1 (2.8%)	2 (4.3%)	0.351
	Performed some of the time	2 (5.3%)	3 (6.8%)		3 (8.3%)	2 (4.3%)	
	Performed most of the time	8 (21.1%)	5 (11.4%)		3 (8.3%)	10 (21.7%)	
	Always performed	27 (71.1%)	34 (77.3%)		29 (80.6%)	32 (69.6)	
The doctor/nurse engages me and my family in the decision making regarding my care.	Not performed	1 (2.6%)	1 (2.3%)	0.769	1 (2.8%)	1 (2.2%)	0.497
	Performed some of the time	5 (13.2%)	3 (6.8%)		3 (8.3%)	5 (10.9%)	
	Performed most of the time	6 (15.8%)	6 (13.6%)		3 (8.3%)	9 (19.6%)	
	Always performed	26 (68.4%)	34 (77.3%)		29 (80.6%)	31 (67.4%)	
I feel that my opinions and concerns matter to the doctor/nurse responsible for my care.	Not performed	1 (2.6%)	2 (4.5%)	0.682	2 (5.6%)	1 (2.2%)	0.742
	Performed some of the time	3 (7.9%)	2 (4.5%)		2 (5.6%)	3 (6.5%)	
	Performed most of the time	4 (10.5)	8 (18.2%)		4 (11.1%)	8 (17.4%)	
	Always performed	30 (78.9%)	32 (72.7%)		28 (77.8%)	34 (73.9%)	
I feel I am a partner in my own treatment and healthcare plan.	Not performed	1 (2.6%)	1 (2.3%)	0.478	1 (2.8%)	1 (2.2%)	0.313
	Performed some of the time	4 (10.5%)	2 (4.5%)		1 (2.8%)	5 (10.9%)	
	Performed most of the time	2 (5.3%)	6 (13.6%)		2 (5.6%)	6 (13%)	
	Always performed	31 (81.6%)	35 (79.5%)		32 (88.9%)	34 (73.9%)	

Table 5 shows that about 68% of physicians and nurses indicated that addressing patients directly was extremely important, and 75% of considered that introducing their role was extremely important. In addition, more than one-third believes that patient engagement improved healthcare outcomes. However, about 7% believed that improving healthcare outcomes via patient engagement was not important or not extremely important. No statistical significance difference between physicians’ and nurses’ responses was apparent.

**Table 5:** Tabulation of physicians’ and nurses’ responses regarding patients engagement in care plan

		Position		Total	*P* - value
Scale Items	Level	Nurses(*n* = 64)	Physicians (*n* = 36)		
1. Addressing the patient directly.	Extremely not important	1 (1.6%)	1 (2.8%)	2 (2.0%)	0.586
	Not important	3 (4.7%)	1 (2.8%)	4 (4.0%)	
	Important	14 (21.9%)	12 (33.3%)	26 (26.0%)	
	Extremely important	46 (71.9%)	22 (61.1%)	68 (68.0%)	
2. Introduce yourself and your role in the patient’s care.	Extremely not important	1 (1.6%)	1 (2.8%)	2 (2.0%)	0.051
	Not important	3 (4.7%)	0 (0.0%)	3 (3.0%)	
	Important	8 (12.5%)	12 (33.3%)	20 (20.0%)	
	Extremely important	52 (81.3%)	23 (63.9%)	75 (75.0%)	
3. Advocate for patient and family involvement in decision making to the extent they choose.	Extremely not important	2 (3.1%)	2 (5.6%)	4 (4.0%)	0.563
	Not important	3 (4.7%)	0 (0.0%)	3 (3.0%)	
	Important	17 (26.6%)	10 (27.8%)	27 (27.0%)	
	Extremely important	42 (65.6%)	24 (66.7%)	66 (66.0%)	
4. Actively listen to the patient’s concerns about the treatment plan.	Extremely not important	1 (1.6%)	1 (2.8%)	2 (2.0%)	0.900
	Not important	2 (3.1%)	1 (2.8%)	3 (3.0%)	
	Important	16 (25.0%)	11 (30.6%)	27 (27.0%)	
	Extremely important	45 (70.3%)	23 (63.9%)	68 (68.0%)	
5. Always ask the patient his opinion about major health care treatment decision.	Extremely not important	1 (1.6%)	2 (5.6%)	3 (3.0%)	0.666
	Not important	1 (1.6%)	1 (2.8%)	2 (2.0%)	
	Important	17 (26.6%)	8 (22.2%)	25 (25.0%)	
	Extremely important	45 (70.3%)	25 (69.4%)	70 (70.0%)	
6. Believe that patient engagement improves the healthcare outcomes.	Extremely not important	1 (1.6%)	3 (8.3%)	4 (4.0%)	0.207
	Not important	1 (1.6%)	2 (5.6%)	3 (3.0%)	
	Important	16 (25.0%)	10 (27.8%)	26 (26.0%)	
	Extremely important	46 (71.9%)	21 (58.3%)	67 (67.0%)	

Table 6 reveals there is a significant difference (all *p* < 0.001) in the responses of physicians and nurses compared with those of patients and family members in the extent of the patient–physicians/nurses relationship (items 1 and 2), involvement (items 3 and 4), and partnership and shared leadership (items 5 and 6).

**Table 6:** Comparison of the mean responses scores of physicians and nurses perceptions and patients and family members experiences of patient engagement

		Patients Responses (mean ± SD)					
		Item.1: (2.70 ± 0.661)	Item.2: (2.72 ± 0.573)	Item.3: (2.61 ± 0.766)	Item.4: (2.59 ± 0.769)	Item.5: (2.62 ± 0.764)	Item.5: (2.62 ± 0.764)
Physicians and Nurses Responses (mean ± SD)	Item.1: (3.60 ± 0.667)	< 0.001*					
	Item.2: (3.68 ± 0.634)		< 0.001*				
	Item.3: (3.55 ± 0.744)			< 0.001*			
	Item.4: (3.61 ± 0.650)				< 0.001*		
	Item.5: (3.62 ± 0.678)					< 0.001*	
	Item.6: (3.56 ± 0.743)						< 0.001*

*Statistically significant

Table 7 summarizes the responses of physicians and nurses to questions related to patient engagement under the categories of access, knowledge, health education and empowerment, self-management, and facility themes.

**Table 7:** Major themes identified by physicians and nurses for patients’ engagement in care plan

+ Category: Access	+ Category: Knowledge
Subcategory:	Subcategory:
- Establish rapport and interaction with a health professional:	▪ Patient preferences and values
Quotations:	Quotations:
- *“Creating an environment of the mutual report will facilitate the communication between patients and healthcare providers.”* - *“therapeutic communication and trust.”* - *“speaking patients’ mother language will foster the interaction.”*	- *“Identify and acknowledge patients values about his/her willingness to be involved or not, and how to share information with them.”* - *“The concept of family-centered decision making interact with patients’ autonomy.”* - *“Knowledge of male custody.”*
▪ Understand health care condition/problem:	▪ Concepts combine a patients’ knowledge, skills, and ability
Quotations:	Quotations:
- *“Involve patients and their family in family meetings.”* - *“providing medical reports”* - *“Explain and update patients about any improvement or deterioration in his/her health.”*	- *“The patients telling us, I do not want to know more, contact my family.”* - *“Our patients have recurrent brain strokes, and another neurological disease,* etc. *moreover, this constrains their ability to optimize their engagement.”*
▪ Eliminate barriers:	
Quotations:	
- *“Reduce nurse to patient ratio to provide more time for interaction and engagement.”* - *“Enhance expatriate physicians and nurses Arabic language to make patients’ engagement more interactive.”*	
+ Category: Education and empowerment	+ Category: Self-management
Subcategory:	Subcategory:
▪ Self-motivation and respect differences	Quotations: - *“Patients request to restore their functional activities.”* - “*Enabling patients with the skills to manage their daily activities.”*
Quotations:	
- *“Engagement reflected by high satisfaction.”* - *“Patients’ engagement motivates them to adhere to the care plan.”* - *“Self-monitoring and understanding.”* - *“Respect differences in age, gender and education levels.”*	
▪ Self-direction of daily care activities	
Quotations:	
- *“Involve patients in rehabilitation programs.”* - *“Empower patients to be self-dependents.”*	
▪ Emotional support:	
Quotations:	
- *“Emotional and intellectual support for patients to manage their disabilities.”* - *“Play the role of patients advocate.”* - *“Respect.”*	
▪ Dedication to and adherence to care plan:	
Quotations:	
- *“Involve patients in the care plan.”* - *“Patients should participate in the formatting of the care plan.”*	
+ Category: Facility	
Subcategory:	
Quotations	
- *“Access to the available opportunities to engage in patients’ healthy activities.”* - *Provide the patients with the Supportive environments to comply with healthy behaviors.*	
*“Conduct campaigns about patient’s engagement”.*	

## Discussion

An increasing body of evidence shows that patients who are more engaged enjoy better health outcomes at lower cost compared with those who are less engaged in their care [31]. In this survey of patient engagement in care plans implemented in a leading medical facility of Saudi Arabia, we found s satisfactory level of patient engagement. Although it is very important to engage patients, the physicians’ and nurses’ perceptions could be improved. However, patients and their families’ members basically enjoy a very supportive environment within which to increase active participation in their health care plans.

We found no significant relationship between patients’ demographic characteristics and their views on engagement. Similarly, Dakken et al. found that the characteristics of study participants were not related to engagement in care plans [32]. Goggins et al. (2014) reported that demographic characteristics were not associated with the level of patients’ desire for engagement and participation in decision making [33]. We did find that male patients gave more positive responses, which may indicate that males are more forthcoming when communicating with healthcare providers. However, independent of their demographic characteristics, patients wish to be engaged and involved in decision making [34–36]. This is especially the case for vulnerable patients, who may not be aware of treatment options or of how their vulnerability may affect their decision making and engagement [37].

The numbers of responding nurses and physicians differed, and we found that position made a difference; nurses’ perceptions toward patient engagement were more favorable than were those of physicians perhaps reflecting the historical nurse-patient relationship and the advocacy role played by nurses. Unfortunately, about 14% of physicians reported that patient engagement was “not important” or “not extremely important;” administrators must implementa continuous culture of compliance in terms of actively engaging patients and their families in healthcare plans, supporting healthcare providers with the knowledge and skills needed to ensure active patient engagement. The idea that doctor knows patients’ best interest, and can making decisions (paternalism) must be eradicated from contemporary healthcare systems [38]. Instead, physicians must develop, preserve, and sustain patient engagement [39].

The open-ended question explored the views of physicians and nurses. Interestingly, the first theme identified in terms of patient engagement was “access,”; i.e., the process of building rapport with patients and eliminating barriers to patients’understanding of their health condition and associated problems. This is the cornerstone of the physicians/nurses–patients relationshipthis is the first level of engagement. The second theme, which combines knowledge and skill, was “knowing patients’ values, preferences, and ideas.” The third theme was “education and empowerment,” which is associated with self-motivation, respect for differences, and dedication to (and compliance with) the treatment plan. Informed consent and active participation in the treatment plan constituted the “self-management” theme. The latter themes depended on attainment of the “facility” theme.

Effective communication and respect for patients’ values and preferences allow patients to become informed and involved in the process of decision making, facilitating information disclosure by patients [31]. About 95% of all physicians and nurses reported that actively listening to patients’ concerns was “important” or “extremely important.”

Patient health literacy may be a major limitation in terms of acquiring self-management skills and active participation in care. Health literacy is the capacity to seek, understand, and use health information when participating in decisions [40]. Therefore, patients’ skills and adopt strategies that effectively encourage changes in the healthcare system behavior.

In this study, the significant difference between the physicians’ and nurses’ perceptions of patient engagement reflects the actual lived patient engagement experience and provides an evaluation of the process of patient engagement. Although we validated our survey using standard measures of patient engagement, further work using other psychometrically validated patient engagement scales is necessary. Such scales include the Patient Health Engagement (PHE) scale and the Clinician Support for Patient Activation (CS-PAM) instrument for patients and clinicians.

There are no prior local or regional works on patient engagement with which we can compare our results. This empirical qualitative exploration of the principal themes relevant to patient engagement affords meaningful information supporting future research designed to improve patient engagement in Saudi Arabia.

The generalizability of our findings is limited by the small sample size and by the fact that the work was conducted in a single institution. In addition, the usual risk of inaccurate self-reporting may be in play.

## Conclusion

We assessed patient experiences of engagement with a healthcare service and physicians’ and nurses’ perceptions of that engagement. Most responses were positive. Most physicians and nurses believed that patient engagement improved healthcare outcomes, but a few did not. Patient engagement should be further fostered; medical professionals must be educated toward this end. Healthcare providers should not make assumptions about patients’ best interests; rather, they should make every effort to learn and acknowledge patients’ values and preferences, thus empowering the patients.
